# Chronic Traumatic Giant Macular Hole Repair with Autologous Platelets

**DOI:** 10.7759/cureus.955

**Published:** 2017-01-05

**Authors:** Mircea Coca, Fuad Makkouk, Renata Picciani, Bernard Godley, Ahmed Elkeeb

**Affiliations:** 1 Chicagoland Retinal Consultants; 2 Department of Ophthalmology, University of Texas Medical Branch at Galveston

**Keywords:** macular hole, platelets, trauma, retina

## Abstract

We report on the closure of a chronic posttraumatic giant macular hole. The patient presented with decreased vision in the left eye following blunt trauma 20 years prior. His dilated fundus examination revealed a 3000 um base-diameter full thickness macular hole. Surgical repair was performed with pars plana vitrectomy (PPV), internal limiting membrane peeling and autologous platelet concentrate (APC) injected over the macular hole. At one month follow-up, the macular hole had closed on exam and optical coherence tomography (OCT), and the patient reported subjective visual improvement. To our knowledge, this report presents the first case of a chronic giant macular hole successfully closed after undergoing surgery with adjuvant platelets therapy.

## Introduction

Macular holes are typically idiopathic but are also known to occur after trauma [1]. The majority of macular holes fall between 400 to 750 um in width. Those exceeding 1500 um are rare, typically post-traumatic, and are referred to as giant macular holes (GMH) [2]. It is known that increased size of a macular hole and the duration of its presence are predictors of limited visual acuity (VA) improvement and a poor likelihood of closure [3]. The treatment of choice for large macular holes is surgical with pars plana vitrectomy (PPV), gas tamponade, and internal limiting membrane peeling [4]. Various adjuvant therapies including thrombin, autologous serum, transforming growth factor-beta-2, biological tissue adhesive, and autologous platelet concentrate have been investigated with varying degree of success [5].

The use of these adjuvants in GMH repair has been demonstrated in many studies. The most effective adjuvant is thought to be autologous platelet concentrate (APC), as it has been reported to result in a higher rate of anatomical closure [6].Informed patient consent was obtained for this patient's treatment. No identifying patient information is disclosed in this report.

## Case presentation

**Figure 1:**
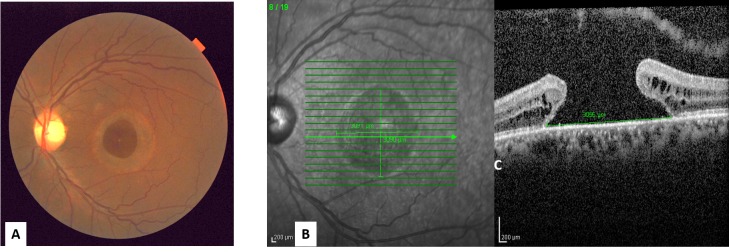
The macular hole at the initial presentation A. The left eye fundus photo shows a large macular hole B. The SD-OCT shows the large full-thickness macular hole

A 50-year-old male presented with 20-years of decreased vision in the left eye after blunt trauma. His best-corrected visual acuity (BCVA) was 20/20 in the right eye and 20/400 in the left eye. Fundus examination revealed a 3000 um base-diameter full thickness macular hole in the left eye (Figure 1 A), confirmed by spectral-domain optical coherence tomography (SD-OCT) (Figure 1 B).

Surgical repair was performed with 23-gauge pars plana vitrectomy, internal limiting membrane peel, autologous platelet concentrate injection over the macular hole, air-fluid exchange, and 12% sulfur hexafluoride (SF6) gas. The APC was prepared as previously described [7].

**Figure 2:**
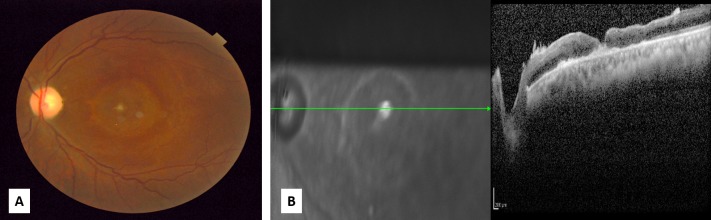
Macular hole at one month post-surgery A. The left eye fundus photo shows the closed macular hole B. SD-OCT confirmed the macular hole has closed

At one month follow-up, SD-OCT confirmed that the macular hole had closed (Figure 2 A, B) and the patient reported subjective improvement with a reduction in the size of his central blind spot. BCVA remained 20/400 in the left eye. The patient continued to report symptomatic improvement of his central blind spot at the two and eight month follow-up visits, as well as some paracentral improvement at 14 months post-surgery. At each follow-up visit the SD-OCT confirmed that the macular hole remained closed.

## Discussion

Traumatic macular holes represent about 10% of all full-thickness macular holes. They are produced by a sudden increase in tangential traction vector forces in the vitreous cortex and exerted on the macula [8]. They are associated with other changes, such as choroidal rupture, contusions of the retinal pigment epithelium, and subretinal hemorrhages. Traumatic macular hole size typically ranges from 150–1500 μm with a mean of about 500 um. Giant macular holes are rare and surgical intervention is not usually considered an option due to poor likelihood of closure [9].

The use of various adjuvants in macular hole surgery has been advocated by numerous authors in order to avoid complications of internal limiting membrane peeling and allow for improved hole closure rates. Although certain studies have advocated that the most effective adjuvant is APC, given the higher rate of anatomical closure, no significant difference in visual outcome has been reported [10]. APC use remains controversial due to the overall success of macular hole closure using the standard approach of pars plana vitrectomy, posterior hyaloid detachment with or without internal limiting membrane peeling, and gas tamponade. However, in patients with a poor likelihood of primary closure, the utility of such a technique needs to be further explored.

Our patient underwent PPV, internal limiting membrane peel, autologous platelet plug injection over the macular hole, and air-fluid exchange. One month later the SD-OCT documented the closure of the macular hole and the patient reported subjective central and peripheral visual improvement throughout the first year despite the BCVA remaining stable at 20/400.

## Conclusions

To our knowledge, our article reports the first case of successful closure of giant chronic macular hole with surgery and adjuvant platelet therapy. Advantages of closing a GMH includes some functional improvement of vision, subjective reduction in the size of the central scotoma, prevention of growth of the hole and reduction in the incidence of macular hole associated retinal detachment.
